# The SWISS IOL Technique (Small-Width Incision Scleral Suture): A Mini-Invasive Technique

**DOI:** 10.1155/2021/8448996

**Published:** 2021-09-13

**Authors:** Mateusz Kecik, Bojan Pajic, Olivier Le Quoy, Gabriele Thumann, Horace Massa

**Affiliations:** ^1^Department of Clinical Neurosciences, Division of Ophthalmology, Geneva University Hospitals, 1205 Geneva, Switzerland; ^2^Faculty of Medicine, University of Geneva, Geneva, Switzerland; ^3^Eye Clinic Orasis, Swiss Eye Research Foundation, 5734 Reinach, Aargau, Switzerland; ^4^Faculty of Sciences, Department of Physics, University of Novi Sad, Trg Dositeja Obradovica 4, 21000 Novi Sad, Serbia; ^5^Fondation Ophtalmologique Adolphe de Rothschild, 75019 Paris, France

## Abstract

**Purpose:**

To evaluate the outcomes and safety of a minimally invasive technique for sutured IOL scleral fixation in case of compromised capsular and iris support.

**Materials and Methods:**

In this retrospective study, we explain our mini-invasive technique and assess the outcomes in terms of visual acuity, pre- or postoperative complications, and IOL position (Sensar AR40e, AMO) in a case series of three patients.

**Results:**

The expected best corrected visual acuity could be achieved after one month. Surgeries were uneventful with a stable eye. No postoperative complications occurred except for one patient who had a conjunctival disinsertion. Neither postoperative hypotony nor raised IOP was found. Additionally, no patient experienced corneal edema at one week control, IOL dislocation, vitreous hemorrhage, or new pupil's irregularity.

**Conclusions:**

In conclusion, each scleral technique has its own advantages and its inherent postoperative complications. To date, there is no evidence of superiority of any single technique. By improving our scleral sutured lens techniques, we could improve peroperative ocular stability, potentially decrease postoperative complication rate, and offer a rapid recovery with a stable visual acuity within a month.

## 1. Should the Sutured Scleral Fixation IOL Technique Be Ostracized? A Mini-Invasive Technique

The ideal place for an intraocular lens (IOL) is in the capsular bag, where it can be tolerated by ocular tissues for decades. Problems arise when the in-the-bag implantation is not feasible. The surgeon has multiple options at this point: sulcus placement, anterior chamber IOL, iris-sutured IOL, or iris-claw IOL, but none of the abovementioned techniques are without potential complications.

The IOL placement in a nonphysiologic anatomical position may result in recurrent iritis, UGH syndrome, ocular hypertension and glaucoma, macular edema, corneal endothelial cell loss and decompensation, retinal detachment, or IOL dislocation. Additionally, many of the abovementioned techniques require large incisions and result in high induced corneal astigmatism.

A different approach of scleral-fixated IOL has been gaining popularity since it was first employed by Maggi and Maggi in 1997 [1]. The major advantages of such placement are that it can be employed irrespective of the iris anatomy and capsular support, closely mirrors the physiological lens position in the eye minimizing aniseikonia, and has a reduced risk of recurrent iritis. Scleral fixation has further evolved in the 21st century with two major techniques emerging: suture fixation and sutureless techniques.

Most techniques require large incisions of 3.0 to 7 mm or scleral flaps leading to eye fragility and instability during surgery and in the immediate postoperative period. This is a raising concern, especially in the elderly population prone to falls, increasing the incidence of ruptured globes with the expulsion of the intraocular content, especially in the cases of previous large scleral incisions or flaps [2, 3].

In this paper, the authors describe their minimally invasive technique of scleral-fixated IOL with suture fixation and present the preliminary outcomes of three patients treated with this technique.

## 2. Materials and Methods

This is a retrospective case series of 3 consecutive cases performed at Geneva University Hospital between November 2020 and March 2021. All patients signed an informed consent for the surgery and for research purposes during follow-up in accordance with the tenets of the Declaration of Helsinki. Data were retrieved from institutional electronic medical records.

Inclusion criteria for scleral-fixated IOL were aphakia with no capsular bag and poor iris support (i.e., iridodonesis or iris defect) and/or poor endothelial cell count (<1000 cells/mm^2^). Exclusion criteria were pregnancy or inability to give informed consent.

### 2.1. Description of the Surgical Technique

**Figure 1:**
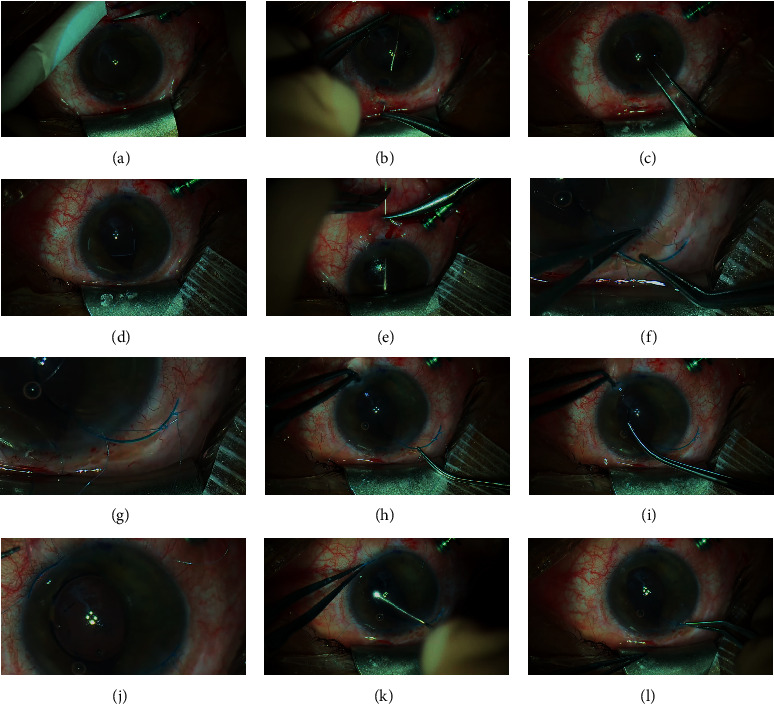
Most important steps of the surgical technique. Intraoperative photographs showing the most important surgical steps (patient 2) (a). Marked sulcus position confirmed with endo-trans-illumination, usually at about 2 mm from the limbus (b). Insertion of the needle passing through the upper sulcus to the contralateral cornea by holding the needle at it basis (c); grasping the 10-0 polypropylene thread with the McPherson forceps, starting with the opposite threads (d), both 10-0 polypropylene threads are passed through the main incision and fixed with stripes superiorly. (e) Needle passing through the lower sulcus to the contralateral cornea by holding the needle with the right hand in the middle and then grasping the needle with the second hand at its basis (f); the right hand is grasping the upper left 10-0 polypropylene thread passed under the haptic, whereas the left hand is pushing the upper right thread of 10-0 polypropylene away (g). Both 10-0 polypropylene threads are well tied on the upper haptic (h). Right hand is maintaining the upper haptic to position the lower haptic in the paracentesis, while the left hand grabs the lower haptic with a small McPherson forceps as close as possible to its extremity (i). Externalization of the lower haptic with McPherson forceps (j). First knot is tied over the lower haptic, starting proximal to the optic (i.e., lower right 10-0 polypropylene) (k). Internalization of the lower haptic: the left hand is holding the extremity of the haptic with a McPherson forceps and pushing it inside in a clockwise movement while the other hand is pushing back the IOL's optic with a vitreous spatula. Same manoeuver with the upper haptic; note the left hand which is pulling on the 10-0 polypropylene to guide the haptic under the iris plane (l).

The appropriate lens power was selected preoperatively using the IOL master 700 and the SRK/T formula with the target of small myopia (−0.25–0.75D). The patient's cornea was marked at the slit lamp at 90° and 270° preoperatively to account for cyclotorsion (Figure 1).

The procedure was performed under general anesthesia.

First, a 23G vitrectomy trocar is placed through the pars plana in the inferior temporal sclera and the infusion is opened at 20 cmH_2_O after confirming its intravitreous placement. Next, an approximately 4 mm conjunctivotomy is performed superiorly and inferiorly with Westcott scissors exposing the bare sclera. Then, the sulcus is located and marked 2 mm from the limbus with gentian violet at 6 and 12 hours (Figure 1(a)). The correct position of the sulcus is confirmed by trans-illumination using an endo-ocular fiber optic illuminator that shows the sulcus as a white line between two darker lines corresponding to the iris root anteriorly and the ciliary body posteriorly.

Next, a clear corneal incision is performed at 120° with a standard angled 2.4 mm phaco knife, which is then slightly enlarged by around 0.5 mm to avoid excessive pressure on the eye during IOL insertion. The IOL is fixated with a double-armed 10-0 polypropylene suture with a STC6 needle. A transcleral passage at one millimeter on each side of the marked limbus on the sclera is necessary. The 10-0 polypropylene threads should stay parallel and go out of the eye through the cornea close to the opposite limbus. To achieve this, the needle should be held as far as possible (Figure 1(b)) and care must be taken in order not to cross the suture. The needles are cut after completion of the transcleral passage. Then, the distant 10-0 polypropylene thread is grasped in the anterior chamber with the McPherson forceps passing over the nearest 10-0 polypropylene thread through the main incision (Figure 1(c)). Once out of the eye, it should be fixed on the left part of the upper eyebrow with a strip. The same procedure should be performed with the another 10-0 polypropylene thread, which should be fixed on the right part of the eyebrow (Figure 1(d)). Again, care must be taken not to cross the threads.

Problems with adequate surgical eye exposure arise when the upper limbus is covered by the upper eyelid when the eye is pushed up. It is then not possible to insert the 10-0 polypropylene holding the STC6 needle at its basis as for the 12 o'clock approach. A useful tip is to use two needle holders: the first one holds the needle in the middle when doing the transcleral insertion, and once it arrives at the sclera, the other needle holder grabs the needle at its base and pushes it down into the conjunctival cul-de-sac (Figure 1(e)). With this maneuver, the needle passes in front of the pupil with a straight eye. A gentle push with some upper counteraction allows the needle to pass easily through the upper part of the cornea.

Next, a nasal paracentesis is performed at 4 o'clock with a 20G curved knife. Usually for more comfort, it is advisable to enlarge it slightly. Then, the lower distal 10-0 polypropylene thread is grasped by using a Synskey hook passing through the side incision over the nearest 10-0 polypropylene thread. It is fixed with a strip on the lower right eyelid. The same maneuver is performed for the other 10-0 polypropylene thread, which is fixed on the lower left eyelid with a strip.

A viscoelastic is then injected into the anterior chamber, and the AR40e lens is injected in front of the iris plane while maintaining the upper haptic outside the main incision.

Several knots (at least 2-1-1-1 knots) are necessary to ensure a good IOL fixation at the level of the haptic, taking care not to cross them. The first knot should always be the closest to the lens optic, i.e., the left superior thread of 10-0 polypropylene for the upper haptic and lower right for the lower haptic. The 10-0 polypropylene thread is passed under the haptic in the direction of the lens (Figure 1(f)). The first knot should be very tight and the other tight enough to block the first knot.

Grasping of the lower haptic might sometimes be difficult. It should be positioned under the side incision (Figure 1(h)). A crocodile 20G curved forceps or a tiny McPherson forceps might be necessary to grasp the lower haptic in front of the pre-iris plane and preiris plane and exteriorize it through paracentesis (Figure 1(i)).

Once the surgical knots are completed, the lower haptic is reintroduced into the anterior chamber with Troutman forceps and the lens is pushed back behind the iris plane with the help of a vitreous spatula (Figure 1(k)). This will help the lower haptic to pass behind the iris plane. Simultaneously, the lower 10-0 polypropylene should be pulled, which allows the lower haptic to be positioned without difficulty at the level of the sulcus (Figure 1(k)).

Then, the upper haptic is then reintroduced into the anterior chamber by using a Troutman or McPherson forceps at its extremity and pushed under the iris plane through the main incision (Figure 1(l)). Sometimes the upper haptic tends to go into the angle. In such a case, a Sinskey hook can be used to pass the haptic under the iris plane. Again, the upper 10-0 polypropylene should be pulled.

Closure of the main incision with 10-0 nylon might be necessary if it is not properly watertight. The eye is then pressurized to 60 cmH_2_O. The exteriorized 10-0 polypropylenes are tied 2 by 2 without exerting too much pressure on the knot. Next, the globe pressure is decreased to more physiological values (20 to 30 cmH2O) and the 10-0 polypropylene threads tend to relax; its ends are then heated with a cautery to make them round.

The 10-0 polypropylene threads are then flattened on the sclera, and the conjunctiva along with the Tenon capsule is closed with 6.0 Vicryl absorbable suture. Next, the viscoelastic is washed from the anterior chamber, the main incision and paracentesis are hydrosutured, and the vitrectomy trocar is removed. Finally, the surgery is finished by an injection of intracameral cefuroxime, as in a standard phacoemulsification.

The patient is discharged on a topical postoperative regimen of ofloxacine drops 4 times a day for one week and tobramycine and dexamethasone drops 4 times a day for one week, which is then decreased by one drop per day every week. At week 4, topical bromfenac 2 times a day is introduced for 2 weeks.

### 2.2. Data Assessment

The following data were assessed: pre- and postoperative visual acuity (Snellen best corrected visual acuity: BCVA), slit lamp examination, intraocular pressure and fundus examination, and Scheimpflug imaging (Pentacam, Oculus, Wetzlar, Germany) at one month. Eyes were observed for modifications in astigmatism magnitude and axis, IOL centration, tilting, and any postoperative complications. Our operations are routinely recorded, and surgery time and technique of all three cases were reviewed after the cases were finished.

Due to the low number of patients in the preliminary study, no statistical analysis was performed.

## 3. Results

Three patients were recruited in this retrospective study, two males (45 and 51 y.o.) and one female (77 y.o.—patient 1). Aphakia resulted from ocular injury in the 2 male subjects and from a complicated cataract surgery for the female patient.

Clinical data such as visual acuity (logMAR), refraction, intraocular pressure with Goldman applanation tonometry, and astigmatism are summarized in Table 1.

Surgical data such as IOL centration, IOL tilting, and surgery time are summarized in Table 2.

**Figure 2:**
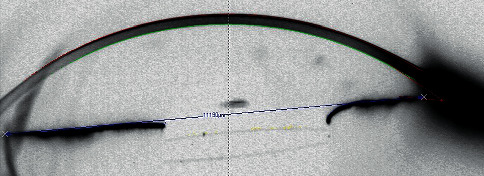
Scheimpflug image of patient 2 in the horizontal meridian with a well-centered IOL and only minor tilt (the blue line connects each angle and is more reliable due to the damaged iris to assess the IOL tilt).

Lenses tilting assessed with the Scheimpflug tomography could retrieve only minor tilting as shown in Figure 2.

They were no peroperative complications such as hypotony, hemorrhage, or eye instability. One patient presented a conjunctival disinsertion of the limbus at week one and required a conjunctival suture in the OR to protect the 10-0 polypropylene threads. No postoperative hypotony, hypertony, corneal edema, IOL dislocation, vitreous hemorrhage, retinal detachment, or new pupil irregularities were noted.

**Figure 3:**
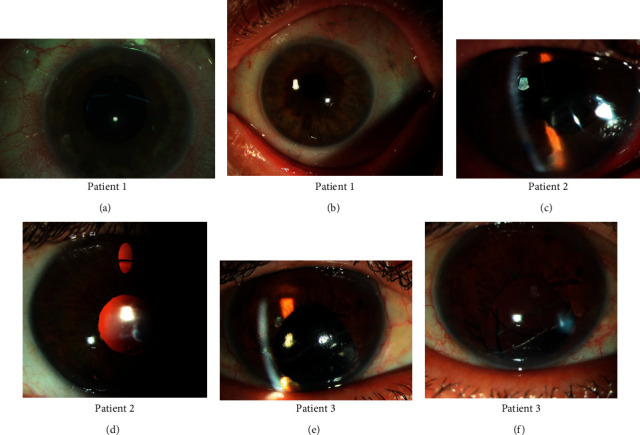
Patient 1: (a) preoperative aphakia with some degree of correctopia and iridodonesis and (b) postoperative slit lamp image; note the 10-0 polypropylene suture visible under the conjunctiva with round ends at 1 and 7 o'clock. Patient 2: (c) preoperative aphakia with remnants of capsular bag and iris sphincter damage and sutured corneal wound and (d) postoperative image with a well-centered IOL as seen through the upper iridotomy. Patient 3: (e) preoperative aphakia, corneal scar, and lower iris sphincter damage with peripheral anterior synechia and (f) well-centered IOL postoperatively. Note the vertical orientation of the lower haptic confirming centration.

Figure 3 shows the pre- and postoperative aspects of all patients.

## 4. Discussion

In our preliminary study, all three consecutive cases recovered visual acuity at one month without the classic complications associated with other scleral fixation techniques. Aphakia correction without a proper capsular bag support remains a challenge, and many different approaches exist. Malbran et al. reported 3 techniques with scleral sutures [4], but they had disadvantages such as suture breaks and cheese wiring. Later, a sutureless approach was developed, albeit with its own set of complications [5, 6]. We have developed a technique of scleral fixation with 2 10-0 polypropylene sutures with a trans-scleral approach and a 6 mm scleral self-sealed incision [7], which could help overcome the 2 main complications of scleral sutured IOLs: suture breaks [8] and cheese wiring effect on the sclera, observed in up to 16% of cases at 7 and 5 years [9]. We have published a case series with more than 20 years follow-up confirming the efficacy of our scleral suture technique [10].

In this paper, we have improved this technique to allow for minimally invasive surgery, small corneal incisions, and fast postoperative recovery.

Visual acuity reached 20/20 at one week in patient 2, whereas the other patients reached their preoperative BCVA at one month. This is an encouraging result when compared to other studies, were BCVA was achieved only after three months [11]. Rapid recovery is mainly due to less eye manipulation and no scleral flap and, in turn, low postoperative inflammation. Visual acuities were limited in patients 1 and 3 due to preoperative optic nerve damage (pituitary adenoma) and posttraumatic macular lesions, respectively.

No complications occurred during surgery, including the absence of ocular hypotony. In comparison, techniques such as sclerotomy and handshake technique require intraoperative haptic extrusion during intrascleral IOL fixation and are usually associated with hypotony or eye instability [12].

Moreover, we did not experience any ocular hypotony in the postoperative period, which is known to occur in intrascleral haptic fixation [13]. This is due to the small corneal incision when compared to our previous technique making it as small as for a standard cataract surgery and to limited transecting of the sclera with one-way passage of the 10.0 polypropylene needle. Yamane et al. had to develop a thin-wall needle to overcome the latter complication with their approach [14]. We had no ocular postoperative bleeding in our series, which might be related to the appropriate localization of the sulcus with trans-illumination, small needle diameter, and finally, the apposition of haptics which plug the holes made in the sclera. Regarding the long-term outcomes and the rising concern about lens opacification, especially with the hydrophilic IOLs, our technique uses the hydrophobic AMO AR40e [15].

In our study, the IOLs were well centered postoperatively without any clinically significant tilt. When considering the haptics of sulcus IOLs, their designed position is within the sulcus with their extremities being curved toward the back and the center of the eye. If they are placed outside of this position, they will exert a counterpressure and, as a result, tilting/decentration of the IOL's optic or haptic extrusion might happen [16, 17]. Placing the haptics in their “physiological” position with 2 threads of 10-0 polypropylene guarantees a central position of the IOL with no tilting or instability. However, those results might not be reproduced if the suture is performed on haptics with an eyelet or with only one suture per haptic [18, 19].

Another advantage of this small incision technique is low induced astigmatism with less than 1 diopter depending on the incision placement on the steep or flat meridian. In a clear corneal approach for angular support IOL or for iris clipped IOL, the induced astigmatism might reach up to 3 diopters [20]. In the example of a Carlevale IOL (Soleko SPA, Pontecorvo, Italy), implanted through a clear corneal incision of 2.75–3.00 mm [21], vision too might be significantly decreased, depending on the preoperative amount of astigmatism and the localization of the steep axis [22].

Finally, one major advantage of this technique is its accessibility in any operating room and its low cost as no special instruments nor any assistant are required.

On the downside, our technique has a learning curve and surgery time substantially decreased from patient 1 to patient 3. Still, an 80 minute surgery is longer than the 54-minute time needed to implant the Carlevale IOL [23]. These time figures are consistent with reports comparing the time necessary for flanged vs. sutured IOLs (20 vs. 50 minutes) [16]. Surgery time could be decreased by fixating the lower haptic with the lens still in the cartridge or even by attaching the suture to both haptics before inserting into the cartridge. Unfortunately, the AR40e manufacturer recommends injecting the implant as soon as possible when loaded into the cartridge or at least within 5 minutes to avoid any risk of IOL damage. Fixing the 10-0 polypropylene on the haptic might also be confusing in this position, and an easy error of crossing of the 10-0 polypropylene might waste all time potentially gained. IOL damage was also described when passing through the injector in the presence of the suture with higher risk of disinsertion [24].

A major limitation of our study is the low number of cases and short follow-up; studies have shown that late IOL dislocation occurs after 3-4 years [8, 25]. However, our technique is a less invasive adaptation of our previous one, published in 2003 with a case series of 50 cases and a mean follow-up of 30 months, proving its safety [7].

Another challenge is the refraction target, which must be improved as we experienced a myopic shift in all cases. This could be partially explained by the irregular shape of the traumatized cornea of patients 2 and 3 [26].

Lastly, the difficulty of our technique is that it requires the utmost surgeon concentration when manipulating the 10-0 polypropylene threads and caution is needed to avoid any suture crossing.

In conclusion, many surgical techniques of IOL implantation exist, with each having its own set of advantages and inherent postoperative complications. To date, there is no evidence of superiority of any single technique [27]. We have improved our scleral sutured lens technique in order to allow for the implantation of an IOL through a 2.45 mm corneal incision and suturing with 4 scleral needle passes. This improves peroperative ocular stability, decreases postoperative complication rate, and offers a rapid recovery with a stable visual acuity within a month.

## Figures and Tables

**Table 1:** Evolution of visual acuity, spherical equivalent, astigmatism, and intraocular pressure in the patient population.

Patient: *n* = 3	Preoperative	Postoperative	Change
Visual acuity (logMAR)	0.22	0.22	0
	0	−0.1	−0.1
	0.9	0.7	−0.2

Refraction in spherical equivalent	+10.25	−2.25	−12.5
	+11	−0.75	−11.75
	+13	−1.25	−14.25

Astigmatism (in diopters/axis in degrees)	−1.5/87°	−2.25/79°	−0.75
	−1.25/116°	−1.5/128	−0.25
	−2.75/180°	−2.75/1°	0

Intraocular pressure (mmHg)	14	16	
	20	14	
	16	14	

**Table 2:** Details of preoperative iris status and postoperative IOL centration and tilt in the patient population.

Patient number	1	2	3
Preoperative iris status	Iridodonesis	Damaged sphincter	Damaged sphincter and anterior synechia
IOL centration	Centered	Centered	Centered
IOL tilt	Minor	Minor	Minor
Surgery time (minutes)	140	90	80

## Data Availability

Raw data are available in the supplementary file.
