# Supplementary material for: The SWISS IOL Technique (Small-Width Incision Scleral Suture): A Mini-Invasive Technique
Source: J Ophthalmol. 2021 Sep 13;2021:8448996. doi: 10.1155/2021/8448996 (PMC8452445; doi:10.1155/2021/8448996)
Supplement: Supplementary Materials — The raw dataset is available in the supplementary file. [file 8448996.f1.docx]

| Patient 1 | Pré | Post |
| --- | --- | --- |
| Age | 77 |  |
| Gender | F |  |
| Acuité visuelle corrigée | 0.6 ac +11/-1.5/87 | 0.6 ac -1.25/-2.25/79 |
| astigmatisme | -1.5/90 | -2.25/80 |
| Per operative iris status | iridodonesis |  |
| IOL centration |  | centered |
| IOL tilting |  | none |
| Surgery time minuts |  | 140 with vitrectomy |
| Complication |  | none |
| TIO | 14 | 16 |

| Patient 2 | Pré | Post |
| --- | --- | --- |
| Age | 45 |  |
| Gender | M |  |
| Acuité visuelle corrigée | 1.0 ac +11.75/-1.25/116 | 1.25 ac -1.5/128 |
| astigmatisme | -1.25 | -1.5 |
| Per operative iris status | Damaged iris sphincter |  |
| IOL centration |  | centered |
| IOL tilting |  | none |
| Surgery time minuts |  | 90 |
| TIO | 20 | 14 |

| Patient 3 | Pré | Post |
| --- | --- | --- |
| Age | 51 |  |
| Gender | M |  |
| Acuité visuelle corrigée | 0.125 ac +14.75/-2.75/180 | 0.2 ac +0.25/-2.75/1 |
| astigmatisme | -2.75 | -2.75 |
| Per operative iris status | Inferior PAS and damaged iris sphincter |  |
| IOL centration |  | Centered |
| IOL tilting |  | None |
| Surgery time minuts |  | 80 |
| TIO | 16 | 14 |
